# Disrupting glutamine metabolic pathways to sensitize gemcitabine-resistant pancreatic cancer

**DOI:** 10.1038/s41598-017-08436-6

**Published:** 2017-08-11

**Authors:** Ru Chen, Lisa A Lai, Yumi Sullivan, Melissa Wong, Lei Wang, Jonah Riddell, Linda Jung, Venu G. Pillarisetty, Teresa A. Brentnall, Sheng Pan

**Affiliations:** 1 Department of Medicine, University of Washington, Seattle, WA 98195 USA; 2 Cell Signaling Technology, Inc, Danvers, MA 01923 USA; 3 Department of Surgery, University of Washington, Seattle, WA 98195 USA

## Abstract

Pancreatic cancer is a lethal disease with poor prognosis. Gemcitabine has been the first line systemic treatment for pancreatic cancer. However, the rapid development of drug resistance has been a major hurdle in gemcitabine therapy leading to unsatisfactory patient outcomes. With the recent renewed understanding of glutamine metabolism involvement in drug resistance and immuno-response, we investigated the anti-tumor effect of a glutamine analog (6-diazo-5-oxo-L-norleucine) as an adjuvant treatment to sensitize chemoresistant pancreatic cancer cells. We demonstrate that disruption of glutamine metabolic pathways improves the efficacy of gemcitabine treatment. Such a disruption induces a cascade of events which impacts glycan biosynthesis through Hexosamine Biosynthesis Pathway (HBP), as well as cellular redox homeostasis, resulting in global changes in protein glycosylation, expression and functional effects. The proteome alterations induced in the resistant cancer cells and the secreted exosomes are intricately associated with the reduction in cell proliferation and the enhancement of cancer cell chemosensitivity. Proteins associated with EGFR signaling, including downstream AKT-mTOR pathways, MAPK pathway, as well as redox enzymes were downregulated in response to disruption of glutamine metabolic pathways.

## Introduction

Pancreatic ductal adenocarcinoma (PDAC) accounts for 80–90% of pancreatic malignancies, and is an aggressive and devastating disease characterized by its late diagnosis, poor prognosis and resistance to chemotherapy^1, 2^. For those patients with non-resectable disease, gemcitabine (GEM) has long been the first-line systemic therapy for the majority of pancreatic cancer patients^3^. However, this drug is highly cytotoxic and the rapid development of innate or adapted drug resistance has been a major hurdle in GEM therapy leading to poor patient outcomes^1, 3, 4^. Therefore, there is a great need to identify drug combinations which can improve the limited efficacy of current pancreatic cancer treatment regimens.

Cancer cells, in comparison to normal cells, have an altered metabolism, including enhanced glycolysis and glutaminolysis. Glutamine is a major nutrient source for many cancer cells, and uptake of glutamine is significantly enhanced in cancer cells along with glucose^5^. The increased aerobic glycolytic activities provide growth advantages to cancer cells by facilitating fast energy generation and supplying metabolic intermediates to be used as building blocks for rapid cell proliferation. As a result, cancer cells are increasingly reliant on glutamine to maintain continuous tricarboxylic acid (TCA) cycle and oxidative phosphorylation in mitochondria. The degree of glutamine dependency could vary among different malignancies. In pancreatic cancer, the cancer cells use a non-canonical glutamine metabolic pathway mediated by oncogenic KRAS to maintain cellular redox state, and such reprogrammed metabolism is required for tumor growth^6, 7^. In addition, glutamine provides an indispensable nitrogen source for glycan biosynthesis through the Hexosamine Biosynthesis Pathway (HBP)^8^, influencing protein glycosylation, maturation and folding. Aberrant protein glycosylation implicated by biosynthesis machinery has long been recognized as a hallmark in epithelial cancers^9, 10^, including PDAC^11^. Emerging evidence has indicated that increased activity of N-glycosylation is implicated in several pancreatic cancer pathways, including TGF-β, TNF, and NF-kappa-B^12^, and inhibition of N-glycosylation can markedly reduce chemoresistance of pancreatic cancer cells^13, 14^. Thus targeting glutamine metabolism could disrupt cancer cell metabolic reprograming in multiple ways and may represent an effective therapeutic approach for PDAC.

One strategy to disrupt glutamine metabolic pathways involves the use of glutamine analogs. 6-diazo-5-oxo-L-norleucine (DON) is a glutamine analog that interferes with both nucleotide and protein synthetic pathways – in which glutamine normally acts as a substrate^15, 16^. The potential anti-cancer activity of DON as a single-agent treatment was previously investigated and showed varied outcomes on different cancer types^15^. Recent data indicated that targeting glutamine metabolism with DON could effectively suppress primary tumor growth and inhibit metastasis in a mouse model of systemic metastasis^17^. In this study, we investigated whether suppression of cancer metabolic pathways through exogenous glutamine analogs would sensitize gemcitabine - resistant pancreatic cancer cells. And further, we sought to elucidate the proteome alterations underlying the cellular physiological changes affected by the disruption of glutamine metabolic pathways.

## Results

### Development of drug resistant pancreatic cancer cell lines

**Figure 1:**
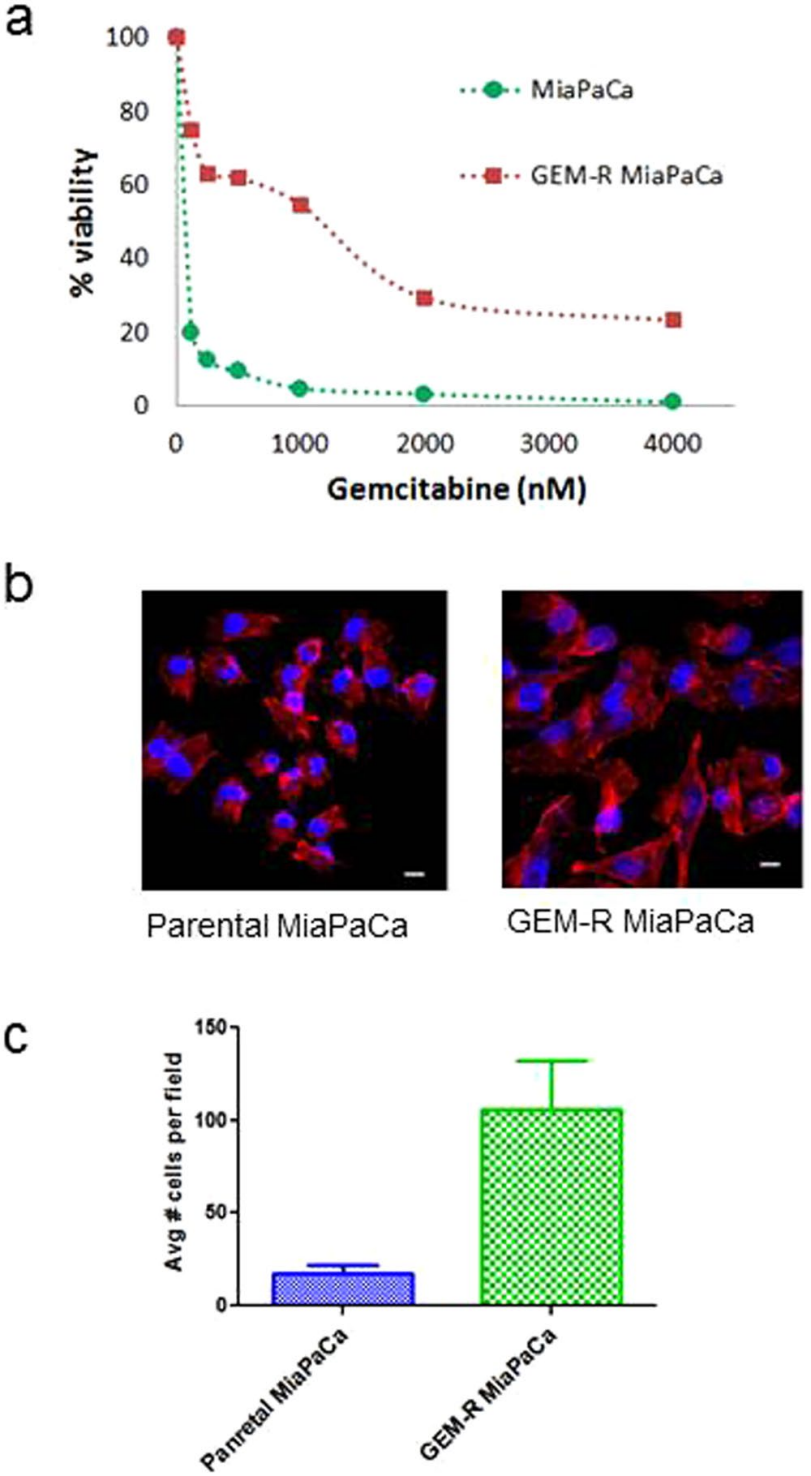
Development of GEM-R MiaPaCa PDAC cells. (**a**) Comparison of GEM-R MiaPaCa cells with the parental MiaPaCa in dose response to GEM treatment. (**b**) Cell morphological images of GEM-R MiaPaCa cells (right) and the parental MiaPaCa cells (left). (**c**) Cell migration assay - the number of cells migrating through the transwell was 4x higher in GEM-R MiaPaCa compared to the parental cells. The error bars represent SDs.

To evaluate if targeting glutamine metabolism could sensitize chemo-resistant PDAC to GEM, we developed and characterized several GEM-resistant (GEM-R) pancreatic cancer cell lines, including GEM-R MiaPaCa and GEM-R HPAF-II. We chose to focus on GEM-R MiaPaCa cells, which was derived from primary PDAC tumor and showed high GEM resistance. GEM-R MiaPaCa pancreatic cancer cells showed sustainable growth and viability in long term culture with 1000 nM gemcitabine whereas parental MiaPaCa pancreatic cancer cells demonstrated IC_50_ of 30–50 nM (Fig. 1a). GEM-R MiaPaCa cells underwent several distinct morphological changes, including increases in cytoplasm and nuclear sizes, and possibly formation of pseudopodia as well (Fig. 1b). These cells also showed significantly increased migration capacity compared to parental MiaPaCa (Fig. 1c). These alterations are similar to the hallmarks of epithelial-to-mesenchymal transition as observed in previous studies^18, 19^. Additional comparisons of viability for GEM-R MiaPaCa to other pancreatic cancer cell lines, as well as normal human pancreatic duct epithelial (HPDE) cells and cancer associated fibroblast cells (CAF) is provided in Supplemental Fig. 1.

### Glutamine disruption effects on cell proliferation and chemosensitivity

**Figure 2:**
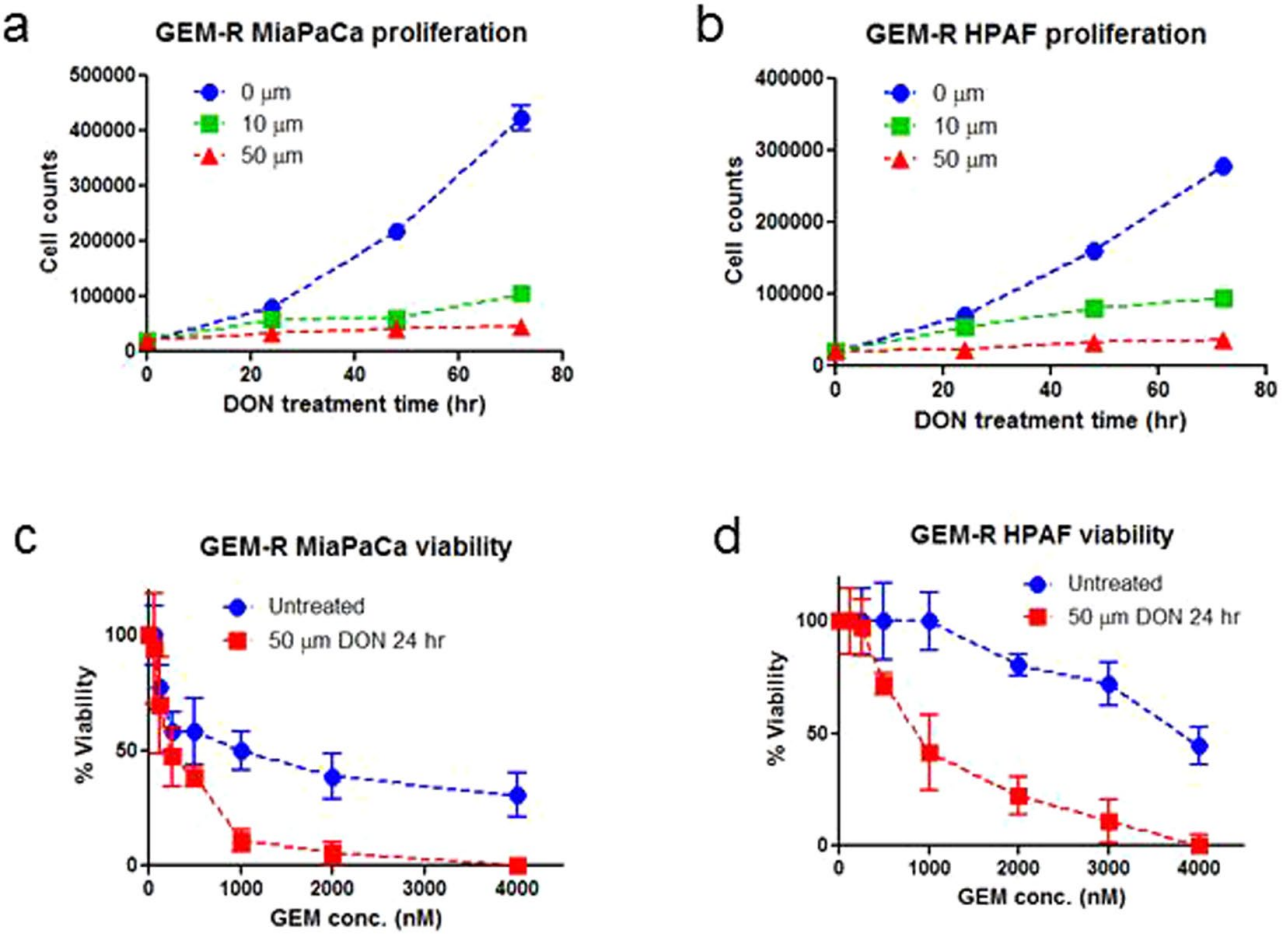
DON treatment of GEM-R pancreatic cancer cells significantly reduced cell proliferation and enhanced chemosensitivity. (**a**) Cell proliferation curves of GEM-R MiaPaCa treated with different concentrations of DON. (**b**) Cell proliferation curves of GEM-R HPAF II treated with different concentration of DON. (**c**) GEM-R MiaPaCa cells treated with 50 µm of DON for 24 hours showed significantly enhanced GEM efficacy. The IC_50_ value decreased 4 fold. (**d**) GEM-R HPAF II cells treated with 50 µm of DON for 24 hours showed significantly enhanced GEM efficacy. The IC_50_ value decreased 4 fold.

The addition of DON to GEM-R MiaPaCa culture resulted in significant differences in cell growth and behavior when compared to untreated cells. 50 µm of DON treatment resulted in an immediate and nearly complete inhibition of GEM-R MiaCaPa proliferation that was sustained over several days (Fig. 2a). Treatment of cells with 10 µM DON also inhibited proliferation, although to a lesser extent. DON treatment of GEM-R HPAF-II resulted in a similar inhibition of cellular proliferation (Fig. 2b). The cell proliferation plots normalized to untreated cells are also provided in Supplemental Fig. 2.

To evaluate the effectiveness of DON as an adjuvant therapy, we tested whether short term (24 hour) treatment with 50 µm of DON would sensitize GEM-R cells to subsequent treatment with GEM. After 72 hour incubation with GEM, we noticed that a single DON treatment significantly enhanced the chemosensitivity of both GEM-R MiaPaCa and HPAF-II cells, with a 4-fold decrease in IC_50_ (Fig. 2c and d).

**Figure 3:**
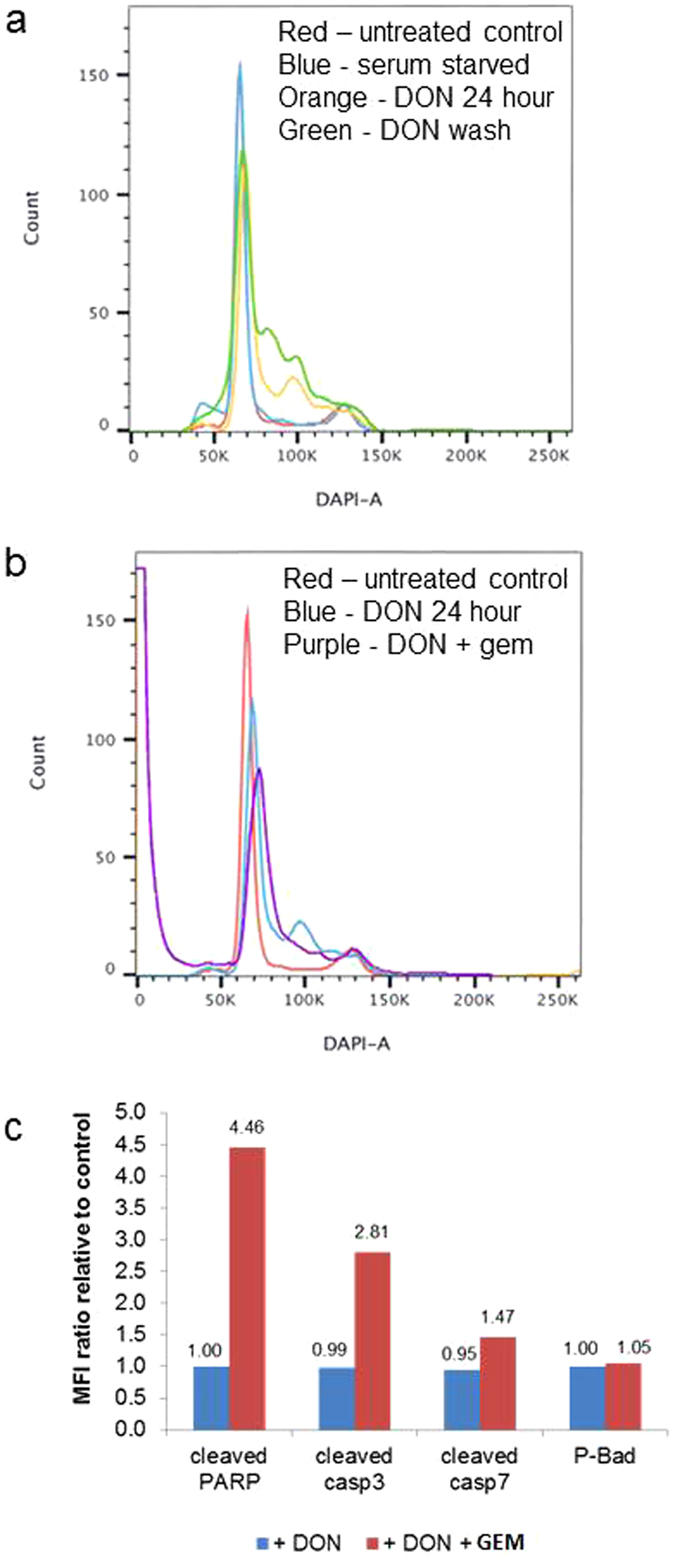
(**a**) Cell cycle analysis of GEM-R MiaPaCa with no treatment (control), incubation with 50 mM DON for 24 hours, exposure to 50 mM DON for 24 hours followed by media change and growth for 24 hours (DON washout). Note the transient G1/S phase block which was reversible following change to fresh media. (**b**) Cell cycle analysis of GEM-R MiaPaCa with no treatment (control), incubation with 50 mM DON for 24 hours, exposure to 50 mM DON for 24 hours followed by 72 hours treatment with GEM. Note the prominent apoptotic peak in the cells treated with DON followed by GEM (purple). (**c**) Multiplex bead based analysis of apoptotic markers, including cleaved PARP, cleaved caspase 3, cleaved caspase 7, and P-Bad, on GEM-R MiaPaCa with incubation with 50 mM DON for 24 hours, sequential DON and GEM treatment using untreated cells (control) as a background.

Flow cytometric analysis showed that DON treatment resulted in a transient G1/S phase block which was reversible following change to fresh media (Fig. 3a). Moreover, we observed a prominent apoptotic sub-G1 peak by flow cytometry in cells treated with DON and gemcitabine (Fig. 3b). Using bead based multiplex assays, we confirmed increases in several apoptotic markers, including cleaved PARP, cleaved caspase 3, and to a lesser extent, cleaved caspase 7 following sequential DON and GEM treatment whereas DON treatment alone showed little changes in these apoptotic markers relative to untreated cells (Fig. 3c).

### Proteome changes associated with RTKs

**Figure 4:**
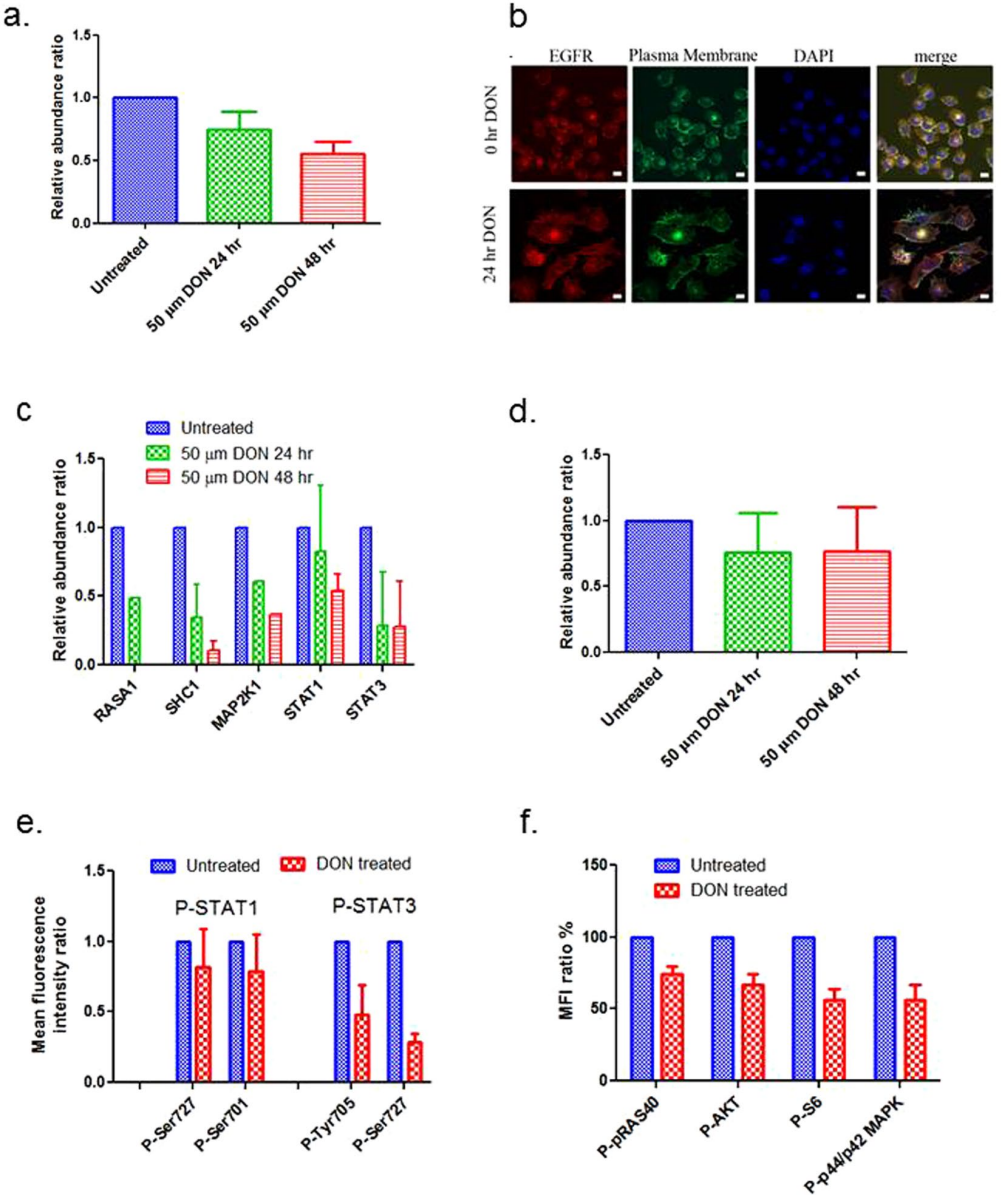
DON treatment influenced the expression and localization of EGFR associated signaling proteins. (**a**) The abundance changes of EGFR in GEM-R MiaPaCa cells before and after treatment with 50 µm of DON (proteomics data). (**b**) Immunofluorescence (IF) images of EGFR in GEM-R MiaPaCa cells before and after DON treatment. red - EGFR, green – plasma membrane, blue - DAPI. (**c**) Abundant changes of the proteins associated with EGFR signaling in MiaPaCa cells before and after DON treatment (proteomics data). (**d**) Expression changes of AKT proteins before and after DON treatment (proteomics data). (**e**) Changes in phospho-STAT1 and phospho-STAT3 before and after DON treatment (50 µm of DON, 24 hr) (multiplex bead based analysis). Two different phosphorylation sites (amino acids are indicated) were measured for each protein. (**f**) Percentage mean fluorescent intensity ratios for phospho-pRas40, phospho-Akt, phospho-S6, and phospho-p44/p42 MAPK for untreated cells (blue) and DON treated cells (red) (multiplex bead based analysis). The error bars represent SDs.

To elucidate the molecular changes associated with glutamine disruption following DON treatment, we performed a dimethyl labeling based quantitative proteomics analysis^20, 21^ to assess the proteome alterations in the GEM-R MiaPaCa cancer cells after DON treatment. The expression profiles of DON treated (24 and 48 hours) GEM-R MiaPaCa cells were compared to untreated GEM-R MiaPaCa cells. Of the 3000 plus proteins that were identified and analyzed, a number of receptor tyrosine kinases (RTKs) proteins were detected, including EGFR, ERBB2, ERBB4, KIT, CSF1R, FLT3, PDGFRB, FGFR1, FGFR2, FGFR3, FLT1, EPHA1, EPHA8, EPHA2, EPHA3, EPHB2, EPHB3. Among them, the epidermal growth factor receptor (EGFR) family has long been associated with drug resistance. EGFR signaling is frequently enhanced in cancer, affecting tumor cell proliferation, angiogenesis, apoptosis, differentiation, and immune responses. A decrease in total EGFR protein levels was detected following 24 and 48 hours DON treatment (Fig. 4a). Immunofluorescence analysis further confirmed a change in the subcellular localization of EGFR. In untreated GEM-R cells, EGFR displayed a predominant membrane localization (Fig. 4b upper panels), whereas following 24 hours of DON treatment, EGFR showed primarily cytoplasmic staining (Fig. 4b lower panels).

The abundances of several other proteins in the EGFR signaling pathway were also decreased following DON treatment (Fig. 4c), suggesting an overall reduction of EGFR signaling. We also noticed that the overall abundance of AKT proteins (AKT1, AKT2 and AKT3 were identified as a protein group in the proteomics analysis) was reduced after treatment with DON (Fig. 4d). AKT proteins are serine/threonine-protein kinases that play key roles in AKT-mTOR pathway, which is downstream of EGFR and implicated in multiple biological processes relevant to drug resistance, including cancer cell metabolism, proliferation, survival and growth^22^.

To confirm the downregulation of EGFR-related signaling pathways, a bead based assay was applied to evaluate changes in phosphorylated kinases/cell signaling pathways in GEM-R MiaPaCa cells following short term treatment with DON. We observed a decrease in phospho-STAT1 and phospho-STAT3 (Fig. 4e). We also observed dramatic drops in phospho-AKT (Ser473) levels as well as phospho-pRas40 (Thr246), phospho-S6 ribosomal protein (Ser 235/236), and phospho-p44/p42 MAPK (Erk1/2) (Thr 202/Tyr204) (Fig. 4f, Supplemental Fig. 3). Ribosomal protein S6 showed a 50% reduction in both phosphorylation and protein expression by flow cytometry and mass spectrometry, respectively. These data provide further evidence that DON impacts downstream signaling that are relevant to drug resistance, possibly through EGFR dependent pathways.

### Effects on glycan biosynthesis

**Figure 5:**
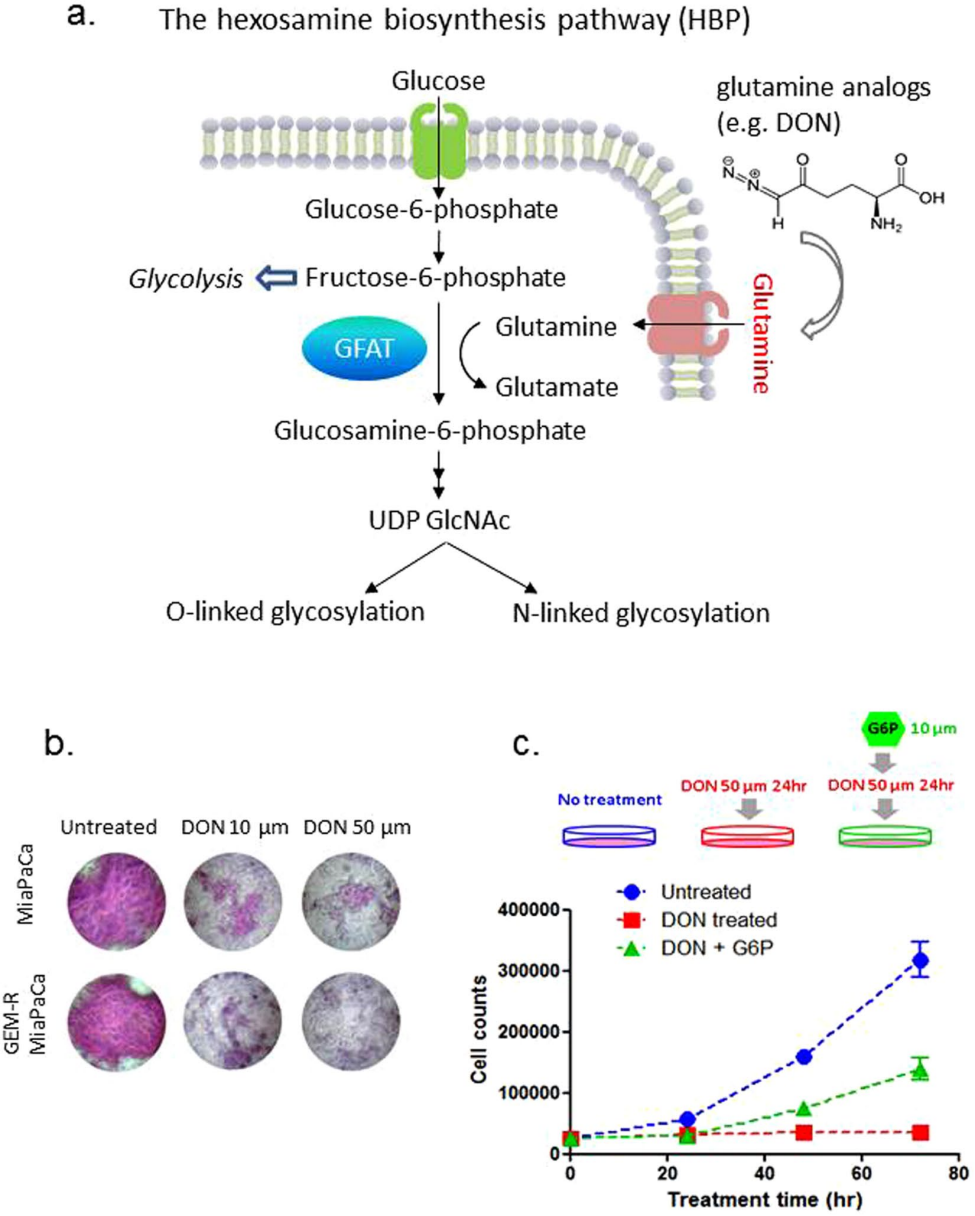
DON treatment impact glycan biosynthesis and contribute to the reduced cell proliferation. (**a**) Disruption of HBP pathway via glutamine analogs impacts glycan biosynthesis. (**b**) PAS staining of GEM-R MiaPaCa cells with no treatment, and with 10 µm and 50 µm of DON treatment for 24 hr. (**c**) “Rescue experiment” - Supplementation of G6P abrogated the inhibition of GEM-R MiaPaCa proliferation. The addition of G6P “rescued” the GEM-R MiaPaCa cells that were treated with DON to ~50% proliferation relative to untreated cells. Blue: untreated cells, Red: cells treated with 50 µm of DON for 24 hours, and Green: cells treated with 50 µm of DON for 24 hours followed by addition of 10 µm of G6P.

Since most RTKs are glycoproteins, the decrease in abundance and loss of membrane localization of EGFR could be, at least in part, explained by the disruption of glycosylation process. One possible link between glutamine disruption and downregulation of EGFR signaling may involve HBP for glycan synthesis (Fig. 5a). HBP synthesizes hexosamines that are used by the endoplasmic reticulum (ER) and Golgi enzymes for protein glycosylation. It is a branch of the glucose metabolic pathway that integrates glutamine metabolism to provide UDP-N-acetylglucosamine (UDP-GlcNAc), the monosaccharide donor molecules for protein glycosylation. A glutamine analog, such as DON, could disrupt HBP by interfering with glutamine:fructose-6-phosphate amidotransferase (GFAT) to suppress UDP-GlcNAc generation, thereby, inhibiting downstream glycosylation.

We first tested for changes in global glycosylation levels following DON treatment. As shown in Fig. 5b, we observed a dramatic decrease in the total level of polysaccharides (PAS staining) after DON treatment. We further demonstrate that inhibition of cellular proliferation is largely abrogated with supplementation of glucosamine-6-phosphate (G6P), the molecule downstream of glutamine and fructose-6-phosphate in HBP. As shown in Fig. 5c, while DON treatment significantly reduced cell proliferation by about 90% compared to the untreated cells, cells that were supplemented with G6P after 24 hours DON treatment were rescued substantially to ~50% proliferation relative to untreated cells. Since G6P does not enter glycolysis pathway and is not available to cells as a fuel source, supplementation of G6P will only replenish the supply of precursors necessary for glycan synthesis, and subsequently protein glycosylation. These results support the model that treatment with the glutamine analog disrupts glycan biosynthesis through the HBP pathway and has a global impact on protein glycosylation and maturation^23^, including the RTK proteins.

### DON treatment impacts cellular redox homeostasis

**Figure 6:**
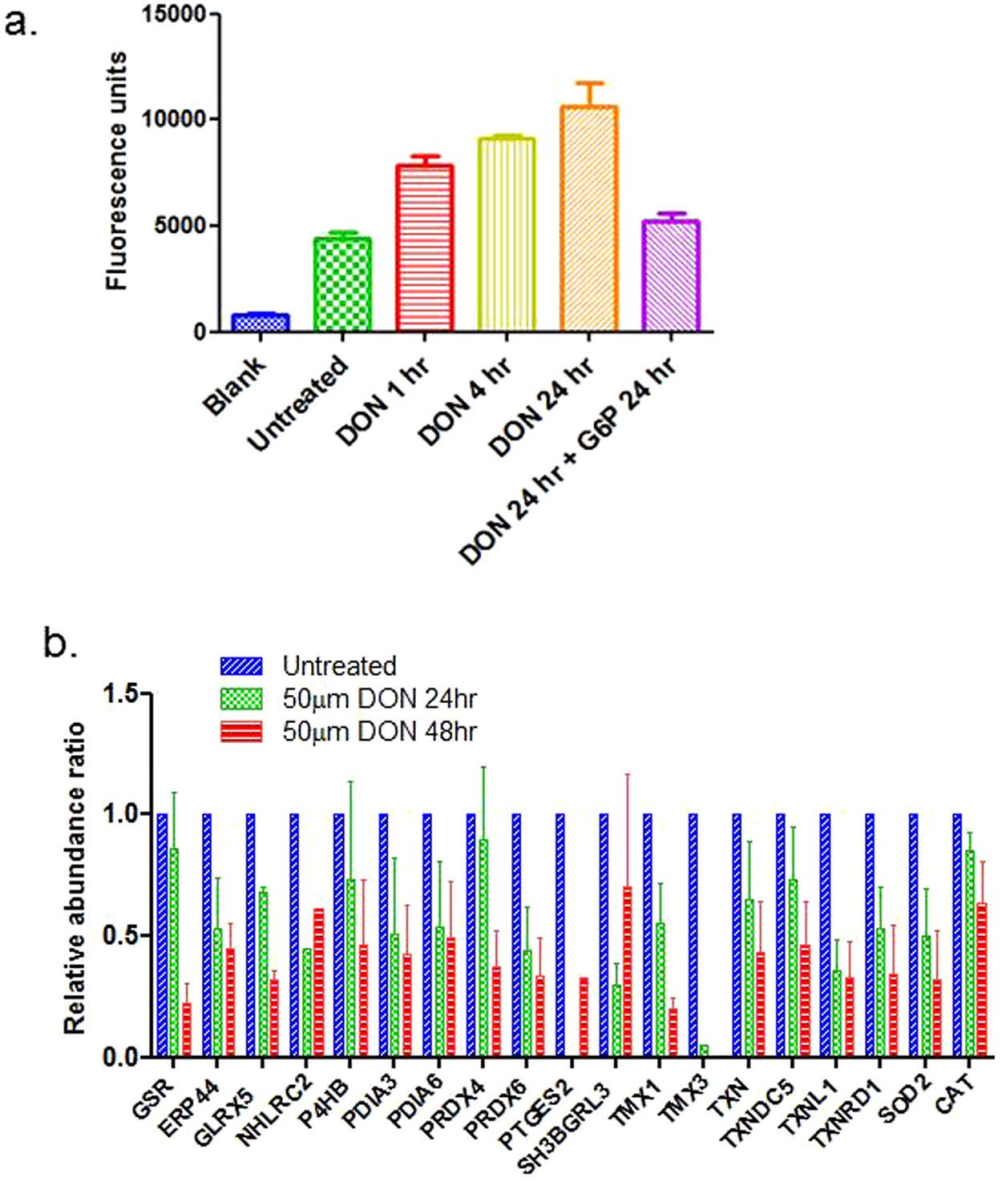
DON treatment imbalanced the redox state of GEM-R MiaPaCa cells. (**a**) GEM-R MiaPaCa cells treated with 50 µm of DON showed increased ROS as the incubation time increased. Addition of G6P reduced the ROS level similar to the untreated cells. (**b**) The expression changes of proteins involved in cell redox homeostasis before and after incubation with 50 µm of DON for 24 hr and 48 hr. The error bars represent SDs.

In addition to its role in the macromolecule biosynthesis and regulation of various signaling pathways, glutamine also participates in maintaining cellular redox homeostasis. To evaluate the redox changes caused by DON treatment, we examined the generation of reactive oxygen species (ROS) in GEM-R MiaCaPa in response to DON treatment. Within one hour of DON treatment, cellular ROS generation was significantly increased (Fig. 6a). By 24 hours of treatment, the ROS was increased by more than two-fold compared to the untreated cells. Remarkably, similar to the G6P rescue effect in the proliferation experiment, GEM-R cells treated with G6P concurrent with DON treatment showed ROS levels comparable to untreated cells.

Proteomic data confirmed changes in cellular redox homeostatic proteins following DON treatment as well. Of note, 16 redox-related proteins showed at least a 2-fold decrease expression after 24-hours or 48-hours of DON treatment (Fig. 6b). Glutathione reductase (GSR) is an important enzyme in producing reducing environment in cells and is responsible for cellular antioxidant defense. The expression of GSR was downregulated more than 4-fold after 48 hours of DON treatment. Down-regulation of GSR and other redox homeostasis proteins may lead to cellular redox imbalance. This observation suggests that disruption of glutamine pathways increases the cellular oxidative state and accumulation of harmful ROS, and is consistent with previous findings, in which, cancer cells use a PDAC specific non-canonical pathway to process glutamine to maintain redox balance^6^.

### Other relevant proteome alterations

**Figure 7:**
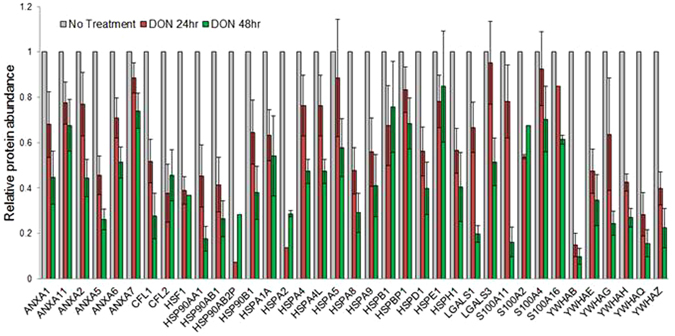
Abundance change of proteins previously associated with chemoresistance of pancreatic cancer, including annexin family, 14-3-3 protein family, cofilins, galectins, S100 proteins and a number of heat shock proteins. The error bars represent SDs.

Notably, several groups of other proteins that have been previously related to drug resistance of pancreatic cancer cells in modulating cancer cell survival, invasiveness and immuno-response^24–32^, were downregulated after DON treatment, including annexin family, 14-3-3 protein family, cofilins, galectins, S100 proteins and a number of heat shock proteins (Fig. 7). The downregulation of these proteins were in favor of reducing chemoresistance of cancer cells. The mechanistic link between the changes of these proteins and DON treatment requires further investigation, and may be related to the cascade of effects resulting from disruption of HBP and other anabolic pathways where glutamine acts as a substrate or precursor. It is also interesting to note that SMAD3, which is activated by TGF-β and has an inhibitory effect on cMYC expression, was significantly up-regulated after DON treatment, suggesting that the treatment might also favor inhibition of proliferation via SMAD3/cMYC axis.

### Proteome changes in exosomes associated with DON treatment

**Figure 8:**
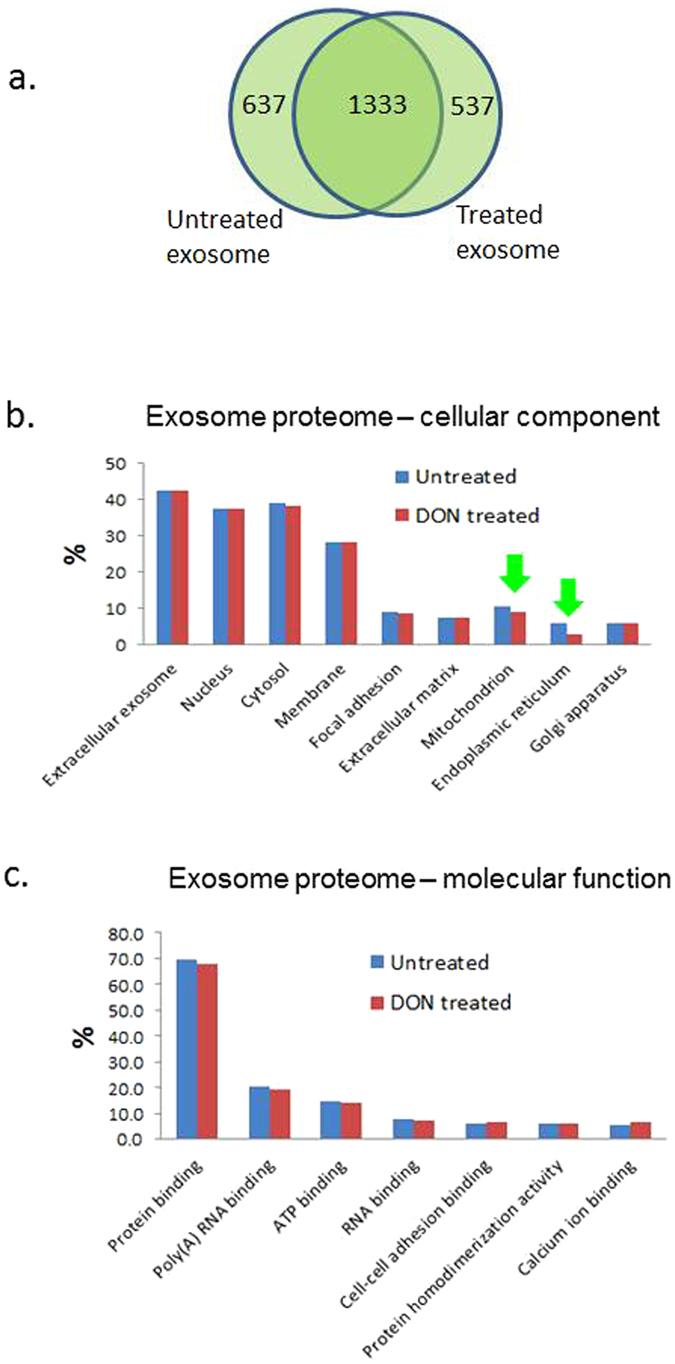
Comparison of protein profiles of exosomes derived from GEM-R MiaPaCa pancreatic cancer cells with and without DON treatment. (**a**) Comparison of protein identification in exosomes derived from GEM-R MiaPaCa cells with and without DON treatment. (**b**) Comparison of cellular location of proteins identified in exosomes derived from GEM-R MiaPaCa cells with and without DON treatment. (**c**) Comparison of molecular function of proteins identified in exosomes derived from GEM-R MiaPaCa cells with and without DON treatment.

Exosomes are enriched with specific proteins, lipids and RNAs^33–35^, and involved in extracellular signaling and chemoresistance of pancreatic cancer cells^36, 37^. Alterations in exosome molecular profiles may reflect the changes of cell functions and physiological states due to perturbations^38^. For the given analytical sensitivity, strikingly, we found that a large portion of the exosome proteome derived from the DON treated GEM-R MiaPaCa cells was not detected in the corresponding intracellular proteome. This portion of the exosome proteins may have a low abundance in cellular proteome, but an increased presence in the extracellular exosome region. We compared the exosome proteomes of the GEM-R MiaPaCa pancreatic cancer cells with and without DON treatment. Altogether >2500 proteins were identified in the exosomes with stringent identification criteria. For both untreated and treated cells, 23 out of the 25 top exosome markers were explicitly identified in their exosome proteome, as listed in Supplemental Table 1, indicating a highly specific and in-depth coverage of exosome profiling. The DON-treated and untreated exosomes showed extended overlap in protein profiles and no significant difference in terms of protein cellular component and molecular function, except the ER proteins (Fig. 8). The number of the ER proteins identified in the exosome derived from the DON treated cells is 2-fold less compared to the untreated exosome (Fig. 8b). This may imply an influence of DON treatment on the ER proteome related to the disruption of glycan synthesis and the sequential glycosylation process, leading to activation of ER stress. We also observed a less extended decrease in the number of mitochondrial proteins, possibly due to the impact on the redox homeostasis. Functional clustering analysis of the unique proteins in the exosome proteomes indicated a significant enrichment of EGF-like domain proteins (p-value 3.05E-11) in the exosomes shed from the DON treated GEM-R MiaPaCa cells (Supplemental Table 2). Remarkably, these 29 proteins were not present in the exosomes shed from the untreated cells. On the other hand, a large number of proteins (n = 88) that are associated with nucleotide binding were only identified in the exosome of the untreated cells, but not in the treated cells. Since the EGF/EGFR signaling is down-regulated in the GEM-R cancer cells that were treated with DON, the enrichment of the EGF-like domain proteins in the exosome may suggest a discharge or re-routing of these proteins to extracellular regions during the glycosylation process possibly due to the disruption of the glycan synthesis pathway. In fact, all 29 EGF-like domain proteins that were solely present in the exosomes secreted from DON treated cells are glycoproteins and most of them have N-linked glycosylations. It is also interesting to note that similar to the EGF-like domain proteins, multidrug resistance protein 1 or P-glycoprotein 1 (ABCB1) – an important membrane glycoprotein mediates drug resistance^39, 40^, was only detected in exosomes isolated from DON treated cells, but not from the untreated cells. P-glycoprotein 1 plays a critical role in drug resistant as an efflux pump to transport many foreign substances (including toxins or drugs) out of cells. The increased presence of P-glycoprotein 1 in the exosomes from DON treated cells, again, may be the result of cellular discharge due to disruption of its glycosylation process.

## Discussion

Several glutamine analog inhibitors including DON have been previously evaluated by *in vitro* studies and early phases clinical trials^41^. However, as a single agent, these analogs only demonstrate limited anti-tumor efficacy. Our results showed that as an adjuvant agent, disruption of glutamine pathways using glutamine analogs can significantly enhance chemosensitivity of drug-resistant pancreatic cancer cells for GEM treatment. GEM resistant pancreatic cancer cells treated with the glutamine analog, DON, showed increased apoptosis and inviability upon treatment with gemcitabine *in vitro*. We have observed that DON treatment results in a transient proliferation arrest, and that this arrest can be abrogated by exposure to G6P. Proteomics analysis showed alterations in EGFR-related and other signaling pathways following DON treatment which were confirmed by cell signaling analysis and immunofluorescence. Our work demonstrates that the anti-cancer effects of glutamine targeting could be mediated through the glycan biosynthesis process, as well as pathways involved in cell redox homeostasis.

Disruption in glycan biosynthesis through HBP could profoundly impact multiple molecular events and signaling pathways associated with drug resistance, including membrane receptors, drug transportation and cellular growth. In HBP, UDP-GlcNAc is an essential substrate for protein glycosylation and plays a role in metabolic homeostasis in cancer progression. Suppression of UDP-GlcNAc could have a cascade of effects on glycan assembly, protein glycosylation, and protein maturation, influencing the expression and functionality of various protein groups, including RTKs, which are mostly transmembrane glycoproteins. Limiting the glutamine supply can not simply reduce UDP-GlcNAc level^42^. However, using glutamine analogs, such as DON, one can interfere with GFAT – the rate limiting enzyme for glycan synthesis, in a controlled manner to disrupt Golgi and cytosolic glycosylation pathways. Our data suggest that DON treatment affects the expression and possibly localization of multiple EGFR signaling proteins, redox enzymes as well as other proteins implicated in drug resistance of pancreatic cancer, leading to the inhibition of cancer cell proliferation and enhancement of chemosensitivity. In fact, most of the EGFR domain proteins have multiple N-linked and/or O-linked glycosylation (EGFR protein has 13 known N-linked glycosylation sites); and AKTs also have multiple O-linked glycosylation sites, which can all be affected by UDP-GlcNAc level that is regulated by HBP. Impairment of protein glycosylation may disrupt protein stability and maturation leading to targeting for ubiquitin-mediated proteasomal degradation of these proteins.

Examination of exosome proteomes revealed a reduction of ER proteins in the exosomes derived from DON treated GEM-R MiaPaCa. Disruption of glycosylation process can disturb ER glycoprotein quality control and result in activation of ER stress, leading to inhibition of protein synthesis and activation of protein degradation^43–45^. Activation of ER stress has been a target for improving efficacy of anticancer therapies^46–48^. The increased presence of EGFR associated proteins and P-glycoprotein 1 in the exosomes from DON treated GEM-R cancer cells compared to the untreated cells, suggests a possible rerouting of these proteins to extracellular region during biosynthesis process due to the impairment on their glycosylation. In cancer treatment, overexpression of EGFR pathway proteins and P-glycoprotein 1 confers resistance to a variety of structurally and functionally diverse anticancer drugs. Disruption of protein glycosylation through HBP pathway on the GEM-R pancreatic cancer cells appeared to counteract metabolic reprogramming by the oncogenes^44, 49^, and effectively down-regulate these protein by impairing their glycosylation process to enhance the chemosensitivity for GEM treatment.

The effects of ROS imbalance as a consequence of chemotherapeutic exposure are complex and perhaps context-dependent. It has both tumor promoting and tumor suppressing effects. In general, cancer cells are known to have elevated level of ROS that contributes to the tumor initiation, promotion and progression, as well as, tumor resistance to chemotherapy^50^. Cancer cells could tolerate the higher level of ROS by having increased activity of antioxidant enzymes. If the ROS level in cancer cells is further escalated to a toxic level, it could result in activation of various cell-death pathways and enhancing chemosensitivity^51^. In fact, a variety of anticancer agents aim to sensitize cancer cells to chemotherapeutic drugs via modulating ROS generation^51^. DON treatment appeared to effectively increase ROS in GEM-R pancreatic cancer cells, in the context of inhibiting Akt, STAT3, and MAPK dependent pathways. DON treatment also resulted in decreased expression of SOD2 and CAT (Fig. 6b), which normally function to remove harmful ROS, and may thereby lower the cellular threshold for tolerating ROS for these cancer cells. It is possible that the disruption of glutamine metabolic processes enhances chemosensitivity to gemcitabine in part through increasing ROS generation and decreasing cellular antioxidant defense. While the exact mechanism and contribution of ROS to DON-induced chemosensitivity remains unclear, our data supports a model whereby disruption of glutamine metabolism leads to a decrease in generation of reducing metabolites and an increase in accumulation of ROS, resulting in a redox imbalance to facilitate apoptosis and cell death in cancer cells.

Our findings suggest that targeting glutamine metabolism using a glutamine analog, such as 6-diazo-5-oxo-L-norleucine, has multifactorial and convoluted influences on HBP, ROS, as well as a number of cascaded cell signaling pathways, and could be an effective therapeutic option to synergize with current GEM therapy. Such an adjuvant treatment can sensitize chemoresistant pancreatic cancer cells by downregulation of EGFR, Akt, and MAPK dependent pathways, as well as modulation of ROS homeostasis. The data presented in this study may help to elucidate glutamine pathway mediated molecular events underlying drug resistance, and facilitate the development of novel adjuvant treatment to enhance the cytotoxicity of GEM towards chemoresistant pancreatic cancer.

## Methods

### Cell lines

MiaPaCa, Panc1 and HPAF cells were obtained from ATCC. HPDE cells were obtained from Dr. Ming-Sound Tsao (University of Toronto, Ontario, Canada) and CAF11-500 cells were a generous gift from Dr. Diane Simeone (University of Michigan). Cancer cells were grown in DMEM or RPMI supplemented with 10% fetal bovine serum and 0.01% penicillin-streptomycin. HPDE cells were grown in keratinocyte media supplemented with EGF and bovine pituitary extract. All cells were maintained in a humidified tissue culture incubator at 37 °C with 5% CO_2_.

### Development of GEM-resistant pancreatic cancer cells

Cells were grown in 6-well dishes in complete DMEM. Gemcitabine was added to 50 nM and gradually increased to 2 µM at 1–2 week intervals.

### Migration Assay

The migration assay was performed as previously described^52^. The lower sides of the transwell inserts (Corning; 8 µm pores) were coated with 100 mg/ml fibronectin overnight at 37 °C. 4 × 10^4^ cells were plated in the upper chamber in serum-free media atop the cells in the lower chamber and incubated for 20 hours. Cells in the upper chamber were removed and inserts were fixed in ice cold methanol. Transwells stained with DAPI, and the number of migrating cells was counted in five random fields. Each sample was run in triplicate and in multiple experiments.

### Cell proliferation assay

Cells were trypsinized from a T-75 flask and counted using a hemacytometer. 25 K cells were inoculated into 24 well dishes. DON and/or gemcitabine was added as indicated. At 24 hour intervals, cells were trypsinized and counted using a hemacytometer. Shown is the average of triplicate wells. Experiment was repeated twice.

### Adjuvant treatment of DON and GEM on GEM-resistant cell lines

Exponentially growing cells were treated with DON +/− gemcitabine as indicated. Gemcitabine was added after 24 hours DON treatment. Fresh media containing DON and/or gemcitabine was added every 24 hours. Cells were trypsinized and counted with a hemacytometer. Shown is the average of triplicate wells.

### ROS assay

Assay was performed using Abcam DCFDA Cellular ROS assay kit (Abcam, Cambridge, MA) as per manufacturer’s instructions.

### Immunofluorescence (IF) staining

Cells were grown in 6-well dishes atop coverslips. Cells were fixed with 4% paraformaldehyde for 10 minutes, washed with PBS, and blocked with 3% BSA. Coverslips were incubated with primary antibody, anti-EGF receptor (Cell Signaling Technology, Danvers, MA) diluted in 1% BSA overnight at 4 °C. Coverslips were washed and incubated with Alexa568 goat anti-rabbit, Cell Mask Plasma Membrane Marker (Thermo Scientific), or Texas Red phalloidin (Invitrogen) for 1 hour at RT in block. Coverslips were washed, fixed for 20 minutes in 4% paraformaldehyde, 0.25 mM glycine for 20 minutes at RT, and mounted onto glass slides with Prolong Gold plus DAPI. Images were taken using a Zeiss LSM 510 Meta confocal microscope with sequential scans using the 568 nm, 488 nm and 405 nm lasers.

### Cell cycle analysis

Cells were trypsinized following no treatment (control), incubation with 50 mM DON for 24 hours, exposure to 50 mM DON for 24 hours followed by media change and growth for 24 hours (DON washout), or exposure to 50 mM DON for 24 hours followed by 72 hours treatment with GEM. Cells were spun down and washed once with 1X PBS. Cell pellets were resuspended in 10 mg/ml DAPI plus 0.1% NP-40 and 10% DMSO and triturated with a 1 ml syringe attached to a 25 G needle to lyse the cells and release intact nuclei. Nuclei were analyzed on a FACScan Canto II. Cell cycle and statistical analyses were performed using FlowJo version 10.

### Cell Signaling analysis

Cell Growth Signaling Multiplex Bead Array kit (Cell Signaling Technology) was used to analyze changes in phospho-pRas 40 (bead 1), phospho-Akt (bead 2), phospho-S6 (bead 3), and phospho-p44/p42 MAPK (bead 4) following DON treatment. Phospho-STAT1 and Phospho-STAT3 kits (Cell Signaling Technology) were used to evaluate P-STAT1 at Ser727 (bead 2) and Tyr701 (bead4) or P-STAT3 at Tyr705 (bead 2) and Ser727 (bead 4). Multiplex Apoptosis Assay kit (Cell Signaling Technology) was used to investigate changes in cleaved PARP, cleaved caspase 3, cleaved caspase 7, and P-Bad. In brief, GEM-R MiaPaCa cells were grown to ~60% confluence. Cells were left untreated, treated with 50 μM DON, or DON followed by GEM for 72 hours. After drug treatment, cells were washed and trypsinized. Protein lysates prepared using the cell lysis buffer (Cell Signaling Technology) supplemented with protease and phosphatase inhibitors. Protein concentrations were measured using BCA assay. 50 ug protein lysate (in 200 µl) was incubated first with 4-plex bead cocktail, then detection antibody cocktail, and finally, streptavidin-PE secondary. Samples were washed and analyzed on the CellSimple Cell Analyzer. Raw data was analyzed using CellSimple software and FlowJo version 10. Scatter plots show one representative experiment. Graphs show the average ratio for three independent experiments.

### Proteomics sample preparation

#### Cell lysate preparation

Cells were trypsinized, collected in 15 ml conical tube, and spun down at 1000 rpm. Supernatant was discarded and cell pellets were rinsed in 1xPBS and spun down again at 1000 rpm. 100–200 µl of M-Per (Thermo Fisher Scientific) with 1x protease inhibitors was added to the cell pellets. Lysates were incubated on ice for 15 minutes and spun at 14,000 rpm for 15 minutes at 4 °C. Supernatant was collected and protein concentration was measured using BCA assay (Thermo Fisher Scientific).

Each lysate sample (1000 μg) was mixed with 5 μg of a protein standard (yeast invertase 2, heat treated at 90–95 °C for 10 min) (Sigma-Aldrich, St. Louis, MO), diluted in 50 mM ammonium bicarbonate solution, and reduced with dithiothreitol (DTT) at 50 °C for 1 hour. The samples were then alkylated with iodoacetamide at room temperature for 30 min in the dark. The samples were purified with TCA precipitation by adding 1/4 volume of 100% w/v trichloroacetic acid. The samples were incubated on ice for 10 min and spun down at 14,000 × g for 5 minutes. Pellets were washed twice with ice cold acetone and air-dried before resuspension in 300 μL of 50 mM sodium bicarbonate solution. Each lysate was digested with sequencing-grade trypsin (Promega, Madison, WI) with a 1:50 trypsin-to-sample ratio at 37 °C for 18 hours.

The digested samples were buffer-exchanged to 100 mM sodium acetate, pH 5.5. Equal amounts of control and diseased sample were separately labeled with formaldehyde-H2 (light) and formaldehyde-D2 (heavy) (Isotec, Champaign, Illinois), respectively. To label each sample, 5 μL of 20% labeling agent was added to a 100 μL sample, immediately followed by the addition of 5 μL of freshly prepared 3 M sodium cyanoborohydride solution. The samples were incubated for 15 minutes at room temperature, with vigorous vortex every few minutes. The light- and heavy-labeled samples were combined and purified through C18 purification columns (the Nest Group, Southborough, MA) following the manufacturer’s instructions.

#### Exosome preparation

Cells were grown in serum-free media for 48 hours and conditioned media was concentrated 20X using an Amicon 3 K centrifugal filter (Millipore, Billerica, MA) as per manufacturer’s instructions. Exosomes were precipitated overnight at 4 °C with ExoQuick TC (System Biosciences, Palo Alto, CA) and spun at 1,500 × g for 30 minutes. Exosome pellet was lysed in RIPA buffer plus protease inhibitors. Protein concentration was measured using BCA assay. The exosome proteins were alkylated, tryptic digested, and C18 purified as aforementioned.

### Mass spectrometry analysis

The dimethyl labeled cell lysate digests were analyzed with an Orbitrap Fusion Tribrid mass spectrometer (Thermo Fisher Scientific) coupled to a nanoAcquity UPLC (Waters, Milford, MA). The samples were first loaded onto a 2 cm IntegraFrit 100 μm trap column (Scientific Instrument Services, Ringoes, NJ) packed with 5 μm/200 Å ProntoSIL C18AQ resin (Mac-Mod Analytical, Chadds Ford, PA) for 10 minutes using 98% Buffer A (D.I. water with 0.1% formic acid) and 2% Buffer B (acetonitrile with 0.1% formic acid) at a flow rate of 2 μL/minute. The peptide samples were then separated by a 25 cm analytical column (75 μm) packed with 5 μm/100 Å ProntoSIL C18AQ resin (Mac-Mod Analytical) using a 90 min linear gradient from 5% to 35% Buffer B versus Buffer A at a flow rate of 300 nL/min. The mass spectrometric analysis was performed using data-dependent acquisition with a m/z range of 400−1600, mass resolution at 120,000, AGC target at 4 × e5 and max injection time of 50 ms for precursor analysis. For MS2 analysis, normalized collision energy of 28 was employed for HCD fragmentation with AGC target at 1 × e4 and max injection time of 50 ms.

The exosome digests were analyzed with an Orbitrap Fusion Lumos mass spectrometer (Thermo Fisher Scientific) using the similar settings except that the MS2 analysis was performed in Orbitrap.

### Proteomics data analysis

The mass spectrometric data was processed using Comet based Trans-Proteomic Pipeline (TPP)^53, 54^. Raw machine output files of MS/MS spectra were converted to mzXML files and searched with comet (2014.02 rev. 2) against the Uniprot human database with the addition of yeast invertase 2. The parameters for database search were as follows: static modifications: carboxamidomethylation on cysteine, light dimethyl on N-terminus and lysine; dynamic modifications: oxidation on methionine, difference between light and heavy dimethyl labeled on N-terminus and lysine. Peptide identifications were assigned probability by PeptideProphet. Only peptides with a FDR ≤ 1% were included for protein identification and quantification. Relative quantitation of heavy and light peptide abundance was performed with Xpress^55^ version 2.1. Proteins present in sample were inferred using ProteinProphet.

Functional annotation of the proteomics data was performed using the Database for Annotation, Visualization and Integrated Discovery (DAVID)^56^ tool from the National Institute of Allergy and Infectious Diseases (NIAID), NIH.

## Electronic supplementary material

Disrupting glutamine metabolic pathways to sensitize gemcitabine-resistant pancreatic cancer
