# Supplementary material for: Disrupting glutamine metabolic pathways to sensitize gemcitabine-resistant pancreatic cancer
Source: Sci Rep. 2017 Aug 11;7:7950. doi: 10.1038/s41598-017-08436-6 (PMC5554139; doi:10.1038/s41598-017-08436-6)
Supplement: Supplementary file 1 — Disrupting glutamine metabolic pathways to sensitize gemcitabine-resistant pancreatic cancer [file 41598_2017_8436_MOESM1_ESM.pdf]

Disrupting glutamine metabolic pathways to sensitize gemcitabine-resistant pancreatic cancer

Ru Chen[1*], Lisa A Lai[1], Yumi Sullivan[1], Melissa Wong[1], Lei Wang[1], Jonah Riddell[2], Linda Jung[2], Venu G. Pillarisetty[3], Teresa A. Brentnall[1], Sheng Pan[1] *

[1]Department of Medicine, University of Washington, Seattle, WA 98195, USA
[2]Cell Signaling Technology, Inc., Danvers, MA 01923
[3]Department of Surgery, University of Washington, Seattle, WA 98195, USA

Supplemental Figure 1. Comparison of GEM viability: GEM-R MiaPaCa, GEM-R Panc, MiaPaCa, Panc, HPDE and CAF.

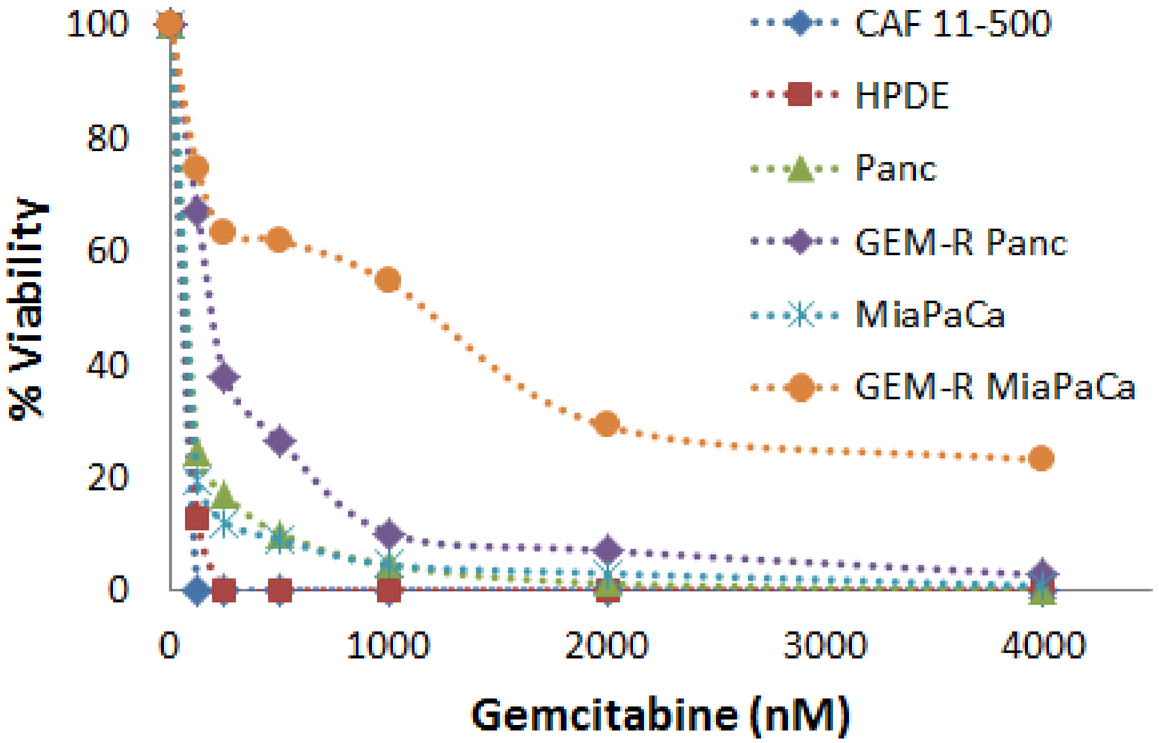

Supplemental Figure 2. Cell proliferation plots normalized to untreated cells.

a. GEM-R MiaPaCa proliferation

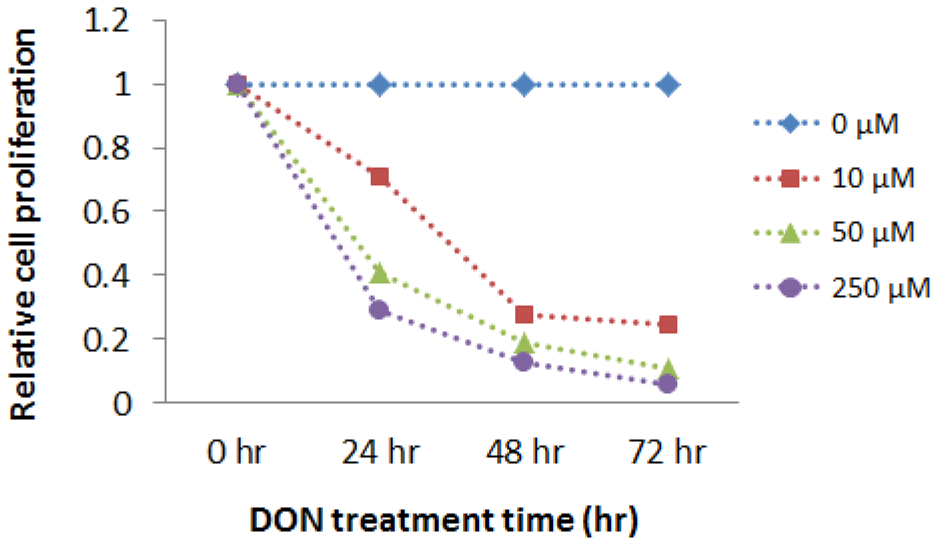

b. GEM-R HPAF proliferation

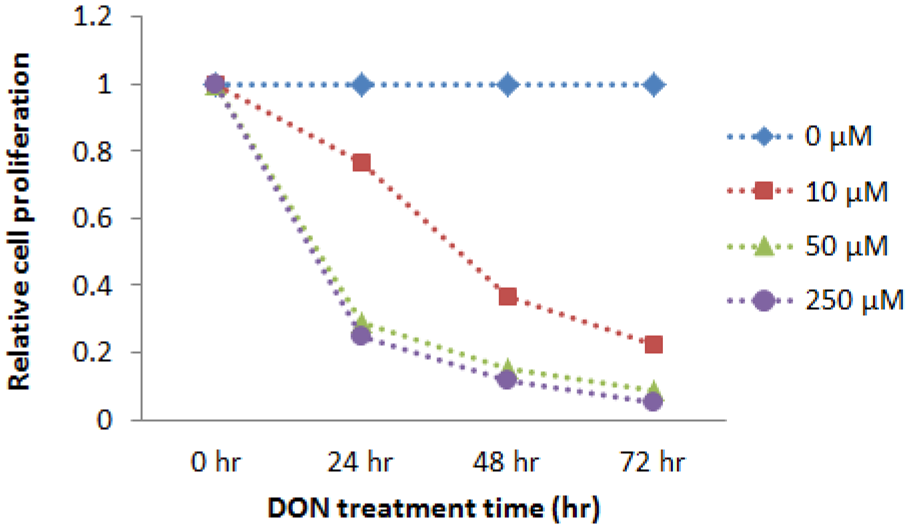

Supplemental Figure 3. Multiplex bead based analysis - Graphs illustrate fluorescence intensity as a function of bead size. Squares reflect gating for individual beads. Bead 1: phospho-pRAS40; bead 2: phospho-Akt; bead 3: phospho-S6; bead 4: phospho-p44/p42 MAPK.

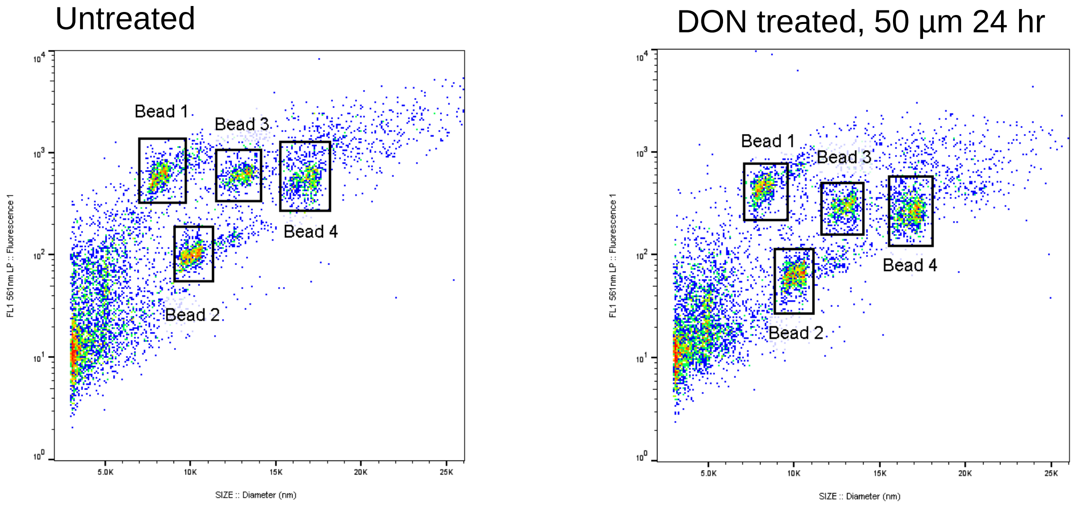

Supplemental Table 1. Identification of top twenty five exosome markers

Name	Spectral count	
	GEM-R	GEM-R DON
HSPA8	45	45
CD9	3	4
GAPDH	115	100
ACTB	98	89
CD63	0	0
CD81	2	2
ANXA2	34	31
ENO1	33	34
HSP90AA1	67	55
EEF1A1	63	50
PKM2	--	--
YWHAE	15	9
SDCBP	6	3
PDCD6IP	11	14
ALB	51	38
YWHAZ	9	7
EEF2	99	97
ACTG1	--	--
LDHA	15	16
HSP90AB1	24	23
ALDOA	24	20
MSN	19	7
ANXA5	9	12
PGK1	34	34
CFL1	11	5

Supplemental Table 2. EGF-like domain proteins significantly enriched in the exosomes secreted from GEM-R MiaPaCa cells treated with DON

Term	PValue	Genes	Fold Enrichment	Bonferroni	Benjamini	FDR
IPR000742:Epidermal growth factor-like domain	3.05E-11	VASN, MATN2, TMEFF2, PEAR1, ATRAID, CD248, ATRN, F7, SCARF1, SLIT2, SCARF2, SLIT3, MUC4, HMCN2, LAMA1, NOTCH1, EYS, FAT3, STAB1, FAT4, FBLN2, TENM2, FAT1, TENM3, HEG1, LRP8, LAMC2, MEGF6, LRP5	4.65	3.22E-08	3.22E-08	4.85E-08
